# Effects of Macleaya Cordata Extract on Performance, Nutrient Apparent Digestibilities, Milk Composition, and Plasma Metabolites of Dairy Goats

**DOI:** 10.3390/ani13040566

**Published:** 2023-02-06

**Authors:** Hao Ling, Hongyan Xiao, Ziyang Zhang, Youkuan He, Peihua Zhang

**Affiliations:** 1 College of Horticulture, Hunan Agricultural University, Changsha 410128, China; 2 Hunan Key Laboratory of Traditional Chinese Veterinary Medicine, Hunan Agricultural University, Changsha 410128, China; 3 College of Animal Science and Technology, Hunan Agricultural University, Changsha 410128, China

**Keywords:** *Macleaya cordata* extract, dairy goats, fatty acid, amino acids, blood physiology, bioactive components

## Abstract

**Simple Summary:**

In this study, we aimed to investigate the effects of *Macleaya cordata* extract (MCE) supplementation on performance, nutrient apparent digestibilities, plasma metabolites, and milk quality in dairy goats. The active ingredients of MCE are sanguinarine and chelerythrine, which are from the medicinal plant (Willd.) R. Br. Dietary supplementation with MCE can increase performance and the apparent nutrient digestibility of fiber in dairy goats.

**Abstract:**

In this study, we aimed to investigate the effects of *Macleaya cordata* extract (MCE) supplementation on performance, nutrient apparent digestibilities, plasma metabolites, and milk quality in dairy goats. Twenty-four lactating Guanzhong dairy goats (*n* = 24) were randomly divided into two groups (each containing 12 goats) in a 52-day trial: the CON group was fed a basal diet; the MCE group was fed a basal diet supplemented with 400 mg/kg MCE. The results indicated that the 4% fat corrected milk yield (4% FCM); uncorrected milk yield; milk-fat concentration; content of C4:0, C18:0, and C18:1n9c fatty acids in milk; and apparent digestibility of neutral detergent fiber (NDF) and acid detergent fiber (ADF) in the MCE group were significantly higher (*p* < 0.05). Furthermore, the lactoferrin (LTF), alpha-lactalbumin (α-La), and beta-lactoglobulin (β-Lg) of the milk and feed conversion rate (FCR) of the goats were significantly greater (*p* < 0.01) in the MCE group than in the CON group. In contrast, the somatic cell count (SCC) (*p* < 0.01), content of C14:0 fatty acids (*p* < 0.01) of milk, and blood urea nitrogen (BUN) concentrations (*p* < 0.05) were significantly lower in the in the MCE goats. These results show that the feeding of MCE can increase the performance and apparent nutrient digestibility of fiber in dairy goats, improving the quality of goat milk.

## 1. Introduction

The dairy goat industry is a significant component of the worldwide dairy industry and is continuously developing; according to the Food and Agriculture Organization of the United Nations’ (FAO) data, in 2017, 220 million dairy goats were stocked worldwide, and goat milk production totaled 18.88 million tons, accounting for 2.3% of total milk production [1]. Simultaneously, the demand for goat milk with more nutritional content and enhanced flavor has surged [2]; according to FAO data, global goat milk consumption maintained an annual increase of at least 10% during the period 2018–2020, with China showing a growth rate of more than 30%. Therefore, there has been a growing emphasis on enhancing the performance and milk quality of dairy goats [3,4,5]. During lactation, the nutritional metabolism of dairy goats is extremely exuberant, making them overly sensitive to the external environment and resulting in them being stress prone (e.g., oxidative damage, inflammation, and rumen bacterial imbalances), both of which affect the health state of the organism, nutrient digestion, and milk production and quality [6]. In the past, subtherapeutic antibiotics were commonly given to animal diets to increase their growth rate, feed intake, and illness resistance, as well as to improve the quality of meat, eggs, and milk [7]. However, starting in 2020, China’s ban on the use of sub-therapeutic antimicrobials in animal production has prompted a rush to discover safer and more effective feed additives with similar effects on antibiotics.

*Macleaya cordata* extract (MCE) is a kind of natural growth promoter [8,9]. Its active chemical constituents are sanguinarine and chelerythrine, extracted from the medicinal plant *macleaya cordata* (Willd.) R. Br. [10]. MCE has anti-inflammatory, antioxidation, sterilization, and anti-tumor properties, which play a role in enhancing animal immunity, promoting animal growth, preventing livestock and poultry diseases, and improving the quality of livestock products [11,12]. For instance, dietary supplementation with MCE alleviated the inflammatory response, improved survival and growth performance in broilers with necrotizing enterocolitis [13], and reduced the inflammatory response in LPS-stimulated porcine jejunocytes [14]. It has been found that MCE attenuates oxidative stress and improves the innate immune response in mice [15], alleviates heat stress in growing pigs and protects against heat-stress-induced increases in colonic permeability [16]. Meanwhile, MCE has been reported to improve growth traits and intestinal health in Salmonella-infected broiler chickens [17] and to enhance performance and improve meat quality as an antibiotic alternative [18]. In addition, MCE can improve the growth performance and intestinal morphology of early weaned piglets, improve the intestinal environment [19], increase the rectal microbial diversity of weaned piglets [20], increase the concentration of essential amino acids in the serum of growing pigs, and improve the growth performance of growing pigs [21]. Furthermore, the addition of MCE to feed can improve the survival and weight-gain rate of grass carp by improving the intestinal morphology, microbiota structure, and mRNA expression levels of intestinal immune-related genes [9]. All these studies indicate that MCE has great potential to promote feeding and improve livestock performance, modulate micro-organisms in the gastrointestinal tract, improve the digestion and absorption of nutrients, and enhance antioxidant function and immunity in animals.

However, the differences in digestion, metabolism, residue remnants, and even performance response to MCE supplementation between species require further research. In particular, studies on MCE in nutrient digestion and absorption metabolism in lactating dairy goats and goat milk quality have not been reported yet. Therefore, the objectives of the present study were to investigate the effects of MCE supplementation on performance, nutrient apparent digestibilities, plasma metabolites, and milk quality in dairy goats.

## 2. Materials and Methods

The animal feeding study was conducted at TAIPING farm (Shaoyang, Hunan, China; latitude 27°14′ N, longitude 111°27′ E).

### 2.1. Animals, Diets and Design

Twenty-four healthy Guanzhong dairy goats with similar daily milk production (1.55 ± 0.12 kg), parity (2.4 ± 0.3), days of milk (192 ± 4.6), and weight (54.96 ± 3.48 kg) were selected and randomly divided into two groups (each containing 12 goats) as follows: control group (CON: fed a basal diet with) and MCE group [fed a basal diet supplemented with 400 mg/kg *Macleaya cordata* extract (Hunan Micolta Bioresource Co., Ltd., Changsha, China, *Macleaya cordata* extract 0.375% *w*/*w* premix)]. The diet was designed in accordance with the Small Ruminant Feeding Standards (NRC 2007) (Table 1).

All of the experimental goats were housed in free stalls with unlimited access to food and water. The goats were milked twice daily (at 6:00 and 19:30) in a 32-by-2 parallel milking parlor. The goats were fed twice daily, at 7:00 and 16:00, with a residual feedstuff content of about 5%. To guarantee that every bit of the supplement was consumed by the goats, 100 g of TMR feed was hand-mixed with MCE before feeding. Subsequently, extra TMR was provided to the goats. The daily milking productivity was recorded using the DelPro milking management program (DeLaval, Stockholm, Sweden). The first 10 days of the trial served as an adaptation period, and the experiment lasted 52 days in total.

### 2.2. Feed and Fecal Sample Collection and Analysis

Dry matter intake (DMI) was determined based on the feed supplied and the orts, with daily feed amounts being recorded. Each goat had weekly feed samples taken, which were then analyzed for DM (method 930.15), CP (method 976.05), EE (method 920.39), calcium and phosphorus (method 935.13), and ash (method 935.13) (method 942.05) [22]. In accordance with Van Soest et al., the amount of neutral detergent fiber (NDF) and acid detergent fiber (ADF) was evaluated. NDF was created using amylase and sodium sulfite [23].

On days 40–42 of the experiment, fresh fecal samples were collected from the rectum of each dairy goat at 8 h intervals in the morning, midday, and evening each day. An amount of 10% sulfuric acid was added for the preservation of ammonia nitrogen (determination of CP) and stored at −20 °C. Finally, all the fecal samples for 3 days from each goat were mixed together according to the serial number of each goat. Then, they were dried at 65 °C, crushed, and passed through a 1 mm sieve to measure DM, CP, EE, NDF, ADF, calcium, phosphorous, and ash, as indicated above. Acid-insoluble ash (AHA) was used as an internal marker to measure nutrient digestibility based on the concentration of AIA in the feed and feces [24].

### 2.3. Milk Sample Collection and Analysis

During the experimental period, the milk yield was recorded daily, and the 4% FCM yield was calculated using Model (1) [25]:

4% FCM = 0.4 × actual milk yield [kg/(head·day)] + 15 × actual milk fat yield [kg/(head·day)](1)

The FCR yield was calculated using Model (2):

FCR = 4%FCM [kg/(head·day)]/DMI [kg/(head·day)](2)

Samples were collected for analysis after 0, 1, 2, 3, 4, 5, 6, and 7 weeks of feeding, with 3 replicate samples being collected for each goat from the flow diverters of the milking machine (9JP-2 × 16, DeLaVel, Stockholm, Sweden) [25]. Milk samples were taken in the morning and evening and blended in a 4:6 ratio based on the amount of milk produced. These samples were split into two centrifuge tubes of 50 mL each. To one of the tubes used to study the composition of the milk, potassium dichromate preservative was added, and the tube was kept at 4 °C. The other tube was utilized for other component analysis and kept at −80 °C for storage.

Analysis of somatic cell count (SCC) (×10,000/mL) in goat milk collected weekly during the trial using a somatic cell analyzer (DeLaval, Stockholm, Sweden).

The content of milk components, fatty acids, amino acids, and bioactive components in the goat milk was analyzed using samples from days 21 and 42. Using a near-infrared reflectance spectroscopic analyzer (Seris300 CombiFOSS; Foss Electric, Hillerd, Denmark), analyses of milk composition were performed. These analyses included measurements of milk fat (%), lactose (%), protein (%), solid no fat (SNF) (%), and urea nitrogen (MUN) (mg/dL).

The fatty acid (FA) composition of the milk samples was analyzed according to the methods of Nguyen et al. [26] and Coppa et al. [27]. Briefly, the milk FA was methylated in accordance with Nguyen et al. [26]. Using an auto-sampler, 0.6 L of the FA methyl esters were fed into a gas chromatograph that had a flame ionization detector (Agilent Technologies 7890A, Wilmington, DE, USA). On a fused silica capillary column with a diameter of 100 mm and 0.25 mm, the FA methyl esters from each sample were separated (CP-Sil 88, Chrompack, Middelburg, The Netherlands). The detector was kept at 260 °C, and the injector temperature was kept at 255 °C. The oven’s initial temperature was held at 70 °C for one minute and then was increased at a rate of 5 °C per minute to 100 °C (held for two minutes), 10 °C per minute to 175 °C (held for 42 min), and 5 °C per minute to a final temperature of 225 °C (held for 15 min). Hydrogen served as the carrier gas, and the analysis was conducted at a pressure of 158.6 kPa. By comparing retention periods to commercially verified standards, peaks were frequently detected. The FA methyl ester proportions were changed to the FA proportions based on the molecular weights of the constituents.

The amino acid composition of the milk samples was analyzed according to Marino et al. [28]. In a nutshell, the HPLC system (Agilent Technologies 1200, Waldbronn, Germany) was used to test sixteen amino acids in the goat milk in duplicate after hydrolyzing them with 6 mol/L HCl at 110 °C for 24 h under vacuum to make common matrix standards.

The lactoferrin (LTF), alpha-lactalbumin (α-La), and beta-lactoglobulin (β-Lg) ELISA kits (Jiansu Meimian Co., Ltd., Yancheng, China) were used to spectrophotometrically (Infinite M Plex, Tecan, Sweden) determine the concentrations of the goat milk LTF, α-La, and β-Lg at a wavelength of 450 nm.

### 2.4. Blood Sample Collection and Analyses

At 21 and 42 days into the study period, jugular venous blood samples from the dairy goats were taken in 10 mL heparin tubes before the morning feeding and centrifuged for 10 min at 3000 g. To prepare the serum for upcoming biochemical index studies, they were separated into 2 mL freezing-storage tubes and kept at 20 °C. The following biochemical blood component analyses were measured by an autoanalyzer (ZY-450, Shanghai Kehua Bio-Engineering Co., Ltd., Shanghai, China): glutamic oxalacetic transaminase (AST), alkaline phosphatase (ALP), total protein (TP), albumin (ALB), triglyceride (TG), glucose (GLU), total cholesterol (TC), high density lipoprotein (HDL), low density lipoprotein (LDL), and serum urea nitrogen (BUN).

### 2.5. Statistical Analysis

The unpaired Student’s *t*-test procedure of SAS 9.4 (SAS Institute Inc., Cary, NC, USA) was used to analyze the effects of *Macleaya cordata* extract on the dry matter intake, milking performance, apparent digestibility of nutrients, somatic cell count (SCC), and milk composition of the dairy goats.

The content of fatty acids, amino acids, and bioactive components in the goat milk and blood index values were analyzed using the SAS 9.4 PROC MIXED procedure. The measurements of samples collected on different sampling dates (days 21 and 42) were considered duplicates, dairy goats within treatment as a random effect, and treatment, time, and interaction of treatment × time as fixed effects. Model (3) is as follows:

Yijk = μ + Ti + Dj + Ti × Dj + Bk + Eijk(3)

Yijk = dependent variable, μ = overall mean, Bk = random effect (1, 2, 3, 4,…, 24 dairy goats within treatment), Ti = treatment effect (CON, MCE), Dj = time effect (21, 42 days), Ti × Dj = interaction of treatment and time, and Eijk = error term.

Data are presented as least squares means with standard errors of the mean. Statistical significance was defined at *p* < 0.05, with highly significant values at *p* < 0.01; trends were declared at 0.05 < *p* < 0.10.

## 3. Results

### 3.1. Effects of MCE on DMI, Lactation Performance, Milk Composition, and Feed Efficiency of Dairy Goats

**Figure 1:**
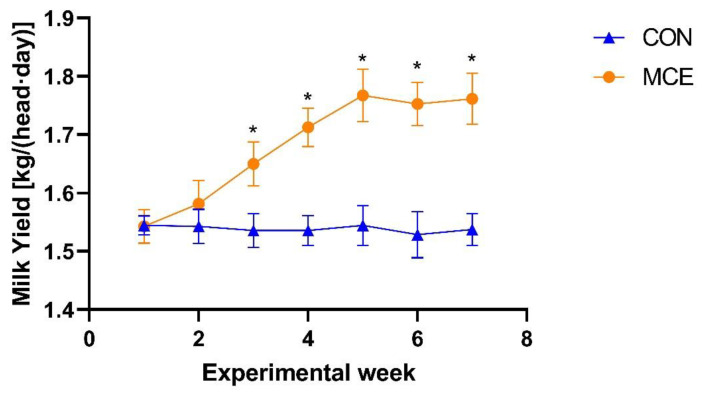
Change in average milk yield throughout the study period in dairy goats. CON—fed a basal diet, MCE—fed a basal diet supplemented with 400 mg/kg macleaya cordata extract. Bars indicate the standard errors. * Superscripts indicate significant difference (*p* < 0.05), while no-sign superscripts indicate no significant difference (*p* > 0.05).

**Figure 2:**
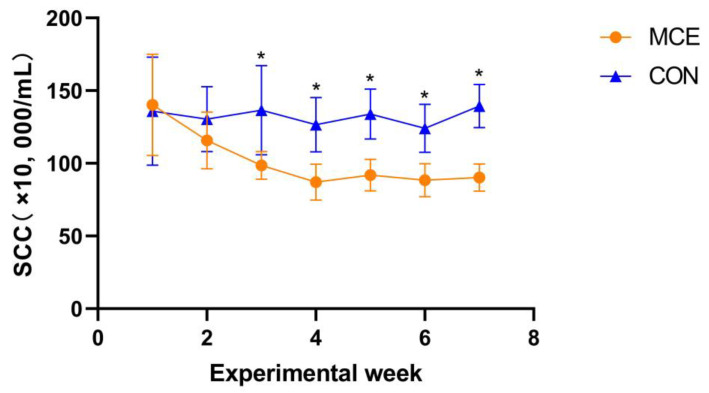
Change in average somatic cell count (SCC) throughout the study period in dairy goats. CON—fed a basal diet, MCE—fed a basal diet supplemented with 400 mg/kg macleaya cordata extract. Bars indicate the standard errors. * Superscripts indicate significant difference (*p* < 0.05), while no-sign superscripts indicate no significant difference (*p* > 0.05).

The DMI did not differ between the CON and MCE groups (*p* > 0.05) (Table 2). The goats fed on a diet supplemented with MCE were observed to have a higher milk yield than the CON group goats from week 3 of the formal trial period to the end of the trial at week 7 (*p* < 0.05) (Figure 1). In addition, as shown in Table 2, the 4% FCM (*p* < 0.01) and milk yield (*p* < 0.05) of the goats in the MCE group were higher than those in the CON group. In terms of milk composition, the milk fat in the MCE group was significantly higher than that of the CON group (*p* < 0.05) (Table 2). As shown in Table 2, the SCC of milk in the MCE group was lower than that in the CON group (*p* < 0.01). Furthermore, the SCC of milk in the MCE group was significantly lower than that of the CON group from week 3 of the formal trial period to the end of the trial at week 7 (*p* < 0.05) (Figure 2). In contrast, there were no significant differences found among the two groups with respect to milk lactose, protein, MUN, and SNF (*p* > 0.05) (Table 2). In the context of feed efficiency, the FCR of goats in the MCE group was significantly higher than that of the CON group (*p* < 0.01) (Table 2).

### 3.2. Effects of MCE on Apparent Digestibility of Nutrients in Dairy Goats

Dietary supplementation with MCE increased the apparent digestibility of NDF and ADF as compared to the CON group goats (*p* < 0.05) (Table 3). However, there were no significant differences between the CON and MCE groups in the apparent digestibility of DM, CP, EE and Ash (*p* > 0.05) (Table 3).

### 3.3. Effect of MCE on Milk FA and Amino Acid Composition

Compared with the CON group, the concentrations of C4:0, C18:0, and C18:1n9c were significantly increased by MCE supplementation (*p* < 0.05) (Table 4). Moreover, compared with the CON group, the MCE group showed significantly lower levels of C14:0 (*p* < 0.01) (Table 4). Meanwhile, compared with the CON group, there was a tendency for the C6:0, C12:0, C14:1, and C16:0 to decrease and the C18:3n3 to increase in the MCE group (0.05 < *p* < 0.1) (Table 4). In addition, the rest of the FAs in the milk did not differ between the two groups (*p* > 0.05) (Table 4).

As shown in Table 5, the two groups showed similar milk amino acid compositions (*p* > 0.05).

### 3.4. Effect of MCE on Milk Bioactive Components

Dietary supplementation with MCE increased the LTF, α-La, and β-Lg in the milk as compared to the CON group (*p* < 0.01) (Table 6).

### 3.5. Effects of MCE on Plasma Metabolites of Dairy Goats

Compared to the CON group, the blood BUN concentrations were lower in goats receiving MCE (*p* < 0.05) (Table 7). Moreover, compared with the CON group, there was a tendency for the AST to decrease in the MCE group (*p* = 0.06) (Table 7). In addition, there were no significant differences in blood parameters reflecting lipid or energy metabolism between the two groups (*p* > 0.05) (Table 7).

## 4. Discussion

As a safe and efficient alternative to feeding antibiotics, MCE has been added to livestock and poultry diets for a long time. Related studies on monogastric animals such as broilers and pigs have shown that MCE can increase feed intake and weight gain, improve the antioxidant and immune functions of the animal organism, and improve intestinal health [17,19,29]. There are relatively few studies related to MCE in ruminants, but there are still studies showing that the addition of MCE to the diet can increase milk production, milk fat production, milk protein production, and lactose production in dairy cows [30]. This is consistent with our findings. In this experiment, the addition of MCE to the diet significantly increased the milk yield and improved the production performance of dairy goats compared to the control group. According to the results of MCE on the apparent digestibility of nutrients in dairy goats in our study (Table 3), it is possible that MCE can modulate the intestinal tissue morphology and microbial community structure of animals [17,19,29] and thus improv the apparent nutrient digestibility, increasing milk production in dairy goats.

Apparent digestibility of nutrients is an important indicator to evaluate the utilization of nutrients in animal diets [31]. Fiber in the diet is digested and broken down to provide energy for rumen micro-organisms and ruminants, which has an important impact on production performance [32]. In this experiment, the addition of MCE to dairy goat diets significantly increased the apparent digestibility of NDF and ADF; some related studies pointed out that NDF and ADF digestibility and milk yield were positively correlated [33], which is in line with our findings. Thus, we assumed that MCE is used to improve milk production in dairy goats by improving the apparent digestibility of NDF and ADF in the diet. Meanwhile, related studies have pointed out that the composition of micro-organisms in the rumen is an important factor affecting the digestibility of NDF and ADF [34]. Therefore, we hypothesized that MCE could improve the digestibility of NDF and ADF by affecting the rumen microbiota of dairy goats, and it will be interesting to further investigate the effect of MCE on the rumen microbiota of dairy goats in the future.

In this experiment, the goat-milk fat rate of the MCE group was significantly higher than that of the CON group. The key precursors for milk fat synthesis are volatile fatty acids such as acetic acid and butyric acid, which are produced by rumen micro-organisms breaking down fiber in the diet [35,36]. Meanwhile, it has been shown that MCE can promote the increase of Lactobacillus spp. flora and enhance the content of butyric acid [8]. Therefore, we speculate that MCE can increase the production of rumen volatile fatty acids by regulating the rumen fermentation characteristics and microbiota of dairy goats, thus increasing the synthesis of milk fat.

SCC is one of the important indicators used to evaluate udder health status and raw milk quality and safety; raw milk quality and health standards in many countries take SCC into account [37,38]. Usually, the SCC and California mastitis test are screening tests for mastitis, and a high SCC indicates poor breast health [39]. Even in healthy dairy goats that are not infected with mastitis, a high SCC can have a negative impact on milk quality and production [40]. In this experiment, the SCC of the MCE group was extremely significantly lower than that of the CON group from the third week to the end of the trial at the seventh week. The vast majority of somatic cells in milk are composed of leukocytes. When inflammation occurs in breast tissue due to bacterial infection or mechanical damage, leukocytes pass through the blood-milk barrier and enter the breast tissue, making SCC increase [41]. MCE has been shown to have excellent antibacterial, anti-inflammatory, immune function-enhancing, and tissue barrier-protective properties [9,15,19,42]. Therefore, we speculate that MCE may reduce SCC by either inhibiting pathogenic microorganisms while alleviating the mammary inflammatory response, or by improving the immune function of animals and protecting the mammary tissue barrier to reduce SCC. The exact mechanism needs to be further explored, but in any case, MCE has great potential to reduce SCC, protect mammary gland health in ruminants, and prevent mastitis.

Fatty acids are closely related to human health and have biological functions such as providing energy, maintaining cell membrane integrity, and preventing cancer, diabetes, and cardiovascular diseases [43]; dairy products are one of the key sources of fatty acids for humans. Currently, no studies have been reported on the regulation of fatty acids in livestock and poultry products by MCE. We found that the addition of MCE to the diet significantly increased the concentration of C4:0 in goat milk. C4:0 is a volatile saturated fatty acid, an important precursor substance for the de novo synthesis of milk fat, and has a variety of biological activities such as providing energy, promoting the development of intestinal lymph nodes and intestinal mucosa proliferation, regulating the body’s immune function, and acting as an antitumor [44]. Some studies point out that C4:0 in milk is mostly created by rumen microbes that digest dietary carbohydrates, which are subsequently taken directly by the stomach lining and collected in the mammary glands [45]. MCE has been shown to promote the growth of lactic acid bacteria and increase the concentration of butyric acid [8]. Therefore, we think that the increased concentration of C4:0 in goat milk may be due to the fact that MCE modulates rumen fermentation characteristics and increases rumen butyric acid yield. Meanwhile, we found that the concentrations of C18:0 and C18:1n9c were higher in the MCE group and lower in C14:0 than in the CON group. This may be due to the modulation of the rumen microbiota by MCE [46], which reduced the biohydrogenation efficiency and increased the proportion of fatty acids leaving the rumen in the diet. The exact mechanism of action of MCE is still unclear and needs more systematic study; however, in this study, we can determine that MCE can regulate the proportion of fatty acid composition in goat milk to some extent and improve milk quality.

Milk protein is one of the main nutrients in milk and is an important indicator for evaluating milk quality [47]. Amino acids are the basic substances for the synthesis of milk protein, and their content and composition ratio determine the quality of milk protein [48]. In the present study, there was no significant difference in amino acid concentrations in goat milk between the MCE and CON groups, which indicates that the addition of MCE to the diet does not negatively affect the amino acid content and composition of goat milk.

LTF, α-La, and β-Lg, all of which are whey proteins, account for about 10~20% of the total protein content in goat milk and have a variety of bioactive functions such as antibacterial functions, antiviral functions, growth promotion, and the regulation of body immunity [49]. In this experiment, the addition of MCE to the diet significantly increased the concentrations of LTF, α-La, and β-Lg in the goat milk. The synthesis of milk protein is closely related to the nitrogen metabolism of the body, and BUN is an important indicator of nitrogen utilization [50]. The nitrogen utilization rate increases when the BUN concentration decreases [51]. We found that the BUN in the MCE group was significantly lower than that in the CON group, which indicates that MCE may increase the concentration of whey proteins such as LTF, α-La, and β-Lg in goat milk by improving nitrogen utilization. 

Serum biochemical indicators are important indicators of the health and physiological metabolism of the animal organism [52]. Serum AST and ALP activities are important indicators of liver function in animals, and the activities of serum AST and ALP increase when liver cells are damaged [53]. In this experiment, the serum AST and ALP activities of dairy goats in the CON and MCE groups were within the normal range, and the differences were not significant, indicating that the addition of MCE to the diet does not negatively affect the liver function of dairy goats. GLU is the main source of energy supply for the body, and the changes in its content can reflect the dynamic balance of the body’s synthesis and metabolism of sugar substances [54]. In this experiment, the serum GLU levels of the dairy goats in the CON and MCE groups were within the normal range and the differences were not significant, so it is assumed that the addition of MCE to the diet did not negatively affect the glucose metabolism of the dairy goats. Serum TP, ALB, and BUN have important nutritional functions which can reflect the absorption and conversion efficiency of protein in the diet and the health status of the animal [25,55]. The BUN content is more sensitive; when its content decreases, it indicates that the efficiency of protein synthesis in the organism is increasing [56]. In this experiment, the serum BUN levels in the MCE group were significantly lower than those in the control group, and the differences in serum ALB and TP levels between the two groups were not significant. So, we presume that the addition of MCE to the diet had a beneficial effect on protein synthesis, conversion, and deposition in dairy goats. Serum TG, TC, HDL, and LDL are important components of blood lipids, and their levels can reflect the status of lipid metabolism in the animal organism [57]. Studies have shown that there are two sources of fatty acids used in milk fat synthesis: first, fatty acids produced by rumen microbial enzymatic lipids or body fat mobilization are used as precursors and are taken up by mammary epithelial cells when they enter the blood circulation and pass through the mammary tissue; second, volatile fatty acids, acetic acid, and β-hydroxybutyric acid, produced by rumen microbial enzymatic carbohydrates, are used as precursors and are directly absorbed by the stomach wall into the mammary tissue [44,58]. In the present experiment, the differences in serum HDL, TG, TC, and LDL content between the CON and MCE groups were not significant. We believe that MCE regulated milk lipid synthesis, probably not by affecting lipid metabolism in the blood but by altering the content of fatty acid synthesis precursors such as rumen acetic acid and butyric acid to affect the ab initio synthesis of the milk lipids.

## 5. Conclusions

This study is the first to analyze the effects of adding MCE to the diets of lactating dairy goats on production performance, nutrient digestibility, and milk quality. Under the conditions of this experiment, the addition of MCE to the diet increased the apparent digestibility of NDF and ADF and increased the milk fat, LTF, α-La, and β-Lg concentrations and milk yield in the dairy goats.

## Figures and Tables

**Table 1:** Chemical composition and nutrient levels of the basal diets (g/kg, DM basis).

Items	Content	Items	Content
Ingredients		Nutrient levels ^b^	
Oat	28	DM	55.17
Alfalfa hay	28	NEL/(MJ/kg)	5.61
Corn	285	CP	16.25
Soybean meal	118.7	NDF	44.53
Wheat bran	20	ADF	24.96
CaHPO_4_	4	EE	4.49
NaHCO_3_	3	Ash	8.82
Limestone	4.9	Ca	0.62
Premix ^a^	4.4	P	0.35
Total	1000		

^a^ The premix provided the following per kg of the diets: VC 10,000 mg, Fe 10,000 mg, Mg 20,000, K 5000 mg, Ca 2000 mg, Zn 10,000 mg, Mn 1000 mg, Cellulose 150,000 U, Xylanase 200,000 U. ^b^ NEL was calculated according to Small Ruminant Feeding Standards (NRC 2007), while other nutrient levels were measured values. VC—vitamin C; DM—dry matter; CP—crude protein; NDF—neutral detergent fiber; ADF—acid detergent fiber; EE—ether extract. Ash—crude ash; Ca—calcium; P—phosphorus.

**Table 2:** Effect of MCE on dry matter intake, milking performance, and milk composition.

Items	Treatment		SEM	*p*-Value
	CON	MCE		
DMI [kg/(head·day)]	2.16	2.13	0.073	0.74
Milking performance				
Milk Yield [kg/(head·day)]	1.54	1.68	0.052	0.04
4%FCM ^a^ [kg/(head·day)]	1.40	1.67	0.049	<0.01
Milk composition				
Fat percentage (%)	3.41	3.99	0.196	0.04
Protein percentage (%)	2.96	2.89	0.073	0.48
Lactose percentage (%)	4.35	4.32	0.029	0.47
SNF (%)	8.00	7.90	0.083	0.37
MUN (mg/dL)	34.95	33.16	0.972	0.28
SCC (×10,000/mL)	132.45	101.75	2.567	<0.01
Feed efficiency				
FCR ^b^ (kg/kg)	0.65	0.78	0.028	<0.01

^a^ 4% FCM = 0.4 × actual milk yield (kg/d) + 15 × actual milk fat yield (kg/d) (NRC, 2007). ^b^ FCR = 4%FCM [kg/(head·day)]/DMI [kg/(head·day)]. CON—fed a basal diet; MCE—fed a basal diet supplemented with 400 mg/kg macleaya cordata extract; SEM—standard error of the mean; SNF—solid no fat; MUN—milk urea nitrogen; SCC—somatic cell count.

**Table 3:** Effects of MCE on apparent digestibility of nutrients (%).

Items	Treatment		SEM	*p*-Value
	CON	MCE		
DM	75.79	78.59	0.399	0.24
CP	72.97	75.52	0.905	0.19
NDF	66.60	70.22	0.712	0.02
ADF	71.58	75.06	0.657	0.01
EE	82.50	85.62	1.400	0.33
Ash	47.03	49.20	1.432	0.42

CON—fed a basal diet; MCE—fed a basal diet supplemented with 400 mg/kg macleaya cordata extract; SEM—standard error of the mean; DM—dry matter; CP—crude protein; NDF—neutral detergent fiber; ADF—acid detergent fiber; EE—ether extract. Ash—crude ash.

**Table 4:** Effect of MCE on milk fatty acid composition (%).

Items	Treatment		SEM	*p*-Value		
	CON	MCE		Treat	Time	Treat × Time
C4:0	1.84	2.16	0.082	0.02	0.39	0.33
C6:0	2.96	3.30	0.118	0.08	0.71	0.15
C8:0	4.63	5.15	0.221	0.16	0.98	0.40
C10:0	16.14	15.95	0.491	0.79	0.90	0.38
C11:0	0.17	0.15	0.008	0.10	0.05	0.05
C12:0	5.14	4.30	0.293	0.08	0.47	0.62
C13:0	0.09	0.08	0.006	0.15	0.31	0.61
C14:0	8.76	7.23	0.344	<0.01	0.50	0.56
C14:1	0.20	0.14	0.018	0.07	0.92	0.41
C15:0	0.79	0.81	0.019	0.55	0.86	0.75
C16:0	24.09	22.51	0.547	0.09	0.68	0.76
C16:1	0.94	0.93	0.030	0.81	0.11	0.89
C17:0	0.53	0.52	0.009	0.32	0.52	0.93
C18:0	7.22	8.38	0.326	0.02	0.72	0.35
C18:1n9t	18.97	19.19	0.425	0.72	0.88	0.28
C18:1n9c	0.30	0.37	0.015	0.01	0.08	0.56
C18:2n6c	4.74	4.93	0.147	0.39	0.12	0.42
C18:3n3	1.38	1.56	0.057	0.08	0.71	0.41
C20:0	0.15	0.16	0.005	0.20	0.82	0.11
C20:4n6	0.28	0.28	0.010	0.91	0.37	0.61
SFA	66.14	64.57	0.657	0.10	0.69	0.18
MUFA	20.38	20.54	0.441	0.79	0.77	0.31
PUFA	6.42	6.84	0.217	0.24	0.17	0.39

CON—fed a basal diet; MCE—fed a basal diet supplemented with 400 mg/kg macleaya cordata extract; SEM—standard error of the mean; SFA—saturated fatty acids; MUFA—monounsaturated fatty acids; PUFA—polyunsaturated fatty acids.

**Table 5:** Effect of MCE on milk amino acid composition (g/100 g).

Items	Treatment		SEM	*p*-Value		
	CON	MCE		Treat	Time	Treat × Time
TAA	2.83	2.78	0.063	0.49	0.78	0.53
Thr	0.14	0.14	0.003	0.71	0.57	0.32
Val	0.22	0.22	0.005	0.84	0.85	0.41
Ile	0.15	0.15	0.003	0.48	0.21	0.98
Leu	0.29	0.28	0.006	0.60	0.34	0.86
Lys	0.25	0.24	0.005	0.40	0.97	0.77
His	0.08	0.08	0.001	0.79	0.70	0.42
Met	0.07	0.07	0.002	0.74	0.49	0.62
Phe	0.15	0.15	0.003	0.58	0.43	0.97
EAA	1.34	1.32	0.030	0.54	0.63	0.89
Asp	0.20	0.19	0.005	0.41	0.90	0.29
Glu	0.55	0.54	0.011	0.70	0.93	0.19
Gly	0.05	0.05	0.002	0.53	0.68	0.44
Arg	0.09	0.08	0.003	0.11	0.74	0.96
Ala	0.09	0.09	0.003	0.30	0.33	0.84
Tyr	0.08	0.08	0.002	0.35	0.03	0.54
Ser	0.13	0.13	0.003	0.33	0.40	0.82
Pro	0.30	0.30	0.006	0.74	0.52	0.37
NEAA	1.49	1.46	0.034	0.46	0.92	0.42

CON—fed a basal diet; MCE—fed a basal diet supplemented with 400 mg/kg macleaya cordata extract; SEM—standard error of the mean; TAA—total amino acids; EAA—essential amino acid; NEAA—nonessential amino acid; g/100 g—concentration per 100 g of true protein.

**Table 6:** Effect of MCE on milk bioactive components (μg/mL).

Items	Treatment		SEM	*p*-Value		
	CON	MCE		Treat	Time	Treat × Time
LTF	152.85	225.13	8.017	<0.01	<0.01	0.93
α-La	1142.62	1591.61	41.721	<0.01	<0.01	0.80
β-Lg	23.68	27.30	0.395	<0.01	<0.01	0.17

CON—fed a basal diet; MCE—fed a basal diet supplemented with 400 mg/kg macleaya cordata extract; SEM—standard error of the mean; LTF—lactoferrin; α-La—alpha-lactalbumin; β-Lg—beta-lactoglobulin.

**Table 7:** Effects of MCE on plasma metabolites.

Items	Treatment		SEM	*p*-Value		
	CON	MCE		Treat	Time	Treat × Time
AST (U/L)	91.58	84.92	2.473	0.06	0.92	0.89
ALP (U/L)	85.33	86.98	3.841	0.76	0.39	0.90
TP (g/L)	79.67	78.92	1.503	0.62	0.15	0.91
ALB (g/L)	48.17	47.58	0.532	0.44	0.08	0.66
TG (mmol/L)	0.21	0.22	0.008	0.37	0.58	0.11
GLU (mmol/L)	2.79	2.90	0.072	0.33	0.01	0.76
TC (mmol/L)	3.13	3.13	0.107	0.97	0.09	0.78
HDL (mmol/L)	2.07	1.99	0.064	0.37	0.19	0.86
LDL (mmol/L)	0.74	0.79	0.033	0.25	0.52	0.73
BUN (mmol/L)	6.31	5.55	0.200	0.03	0.91	0.59

CON—fed a basal diet; MCE—fed a basal diet supplemented with 400 mg/kg macleaya cordata extract.; SEM—standard error of the mean; AST—glutamic oxalacetic transaminase; ALP—alkaline phosphatase; TP—total protein; ALB—albumin; TG—triglyceride; GLU—glucose; TC—total cholesterol; HDL—high density lipoprotein; LDL—low density lipoprotein; BUN—blood urea nitrogen.

## Data Availability

Data are contained within the article.
